# The Application and Mechanism Analysis of Enteral Nutrition in Clinical Management of Chronic Diseases

**DOI:** 10.3390/nu17030450

**Published:** 2025-01-26

**Authors:** Qingye Li, Jing Wang

**Affiliations:** Institute of Food and Nutrition Development, Ministry of Agriculture and Rural Affairs, Beijing 100081, China; liqingye628@163.com

**Keywords:** chronic diseases, enteral nutrition, clinical management, mechanism of action

## Abstract

Chronic diseases have emerged as a significant challenge in global public health due to their complex etiologies, prolonged disease courses, and high treatment costs. With the aging population and changes in lifestyle, the number of patients with chronic diseases has increased dramatically, which has brought heavy burden to families and society. Chronic diseases are often accompanied by digestive and absorptive disorders as well as metabolic disorders, resulting in insufficient nutrient intake, further worsening the condition and weakening the physique. Therefore, the importance of nutritional intervention in chronic disease management has become increasingly prominent. As an important means of nutritional intervention, enteral nutrition plays a key role in improving the nutritional status of patients, promoting rehabilitation, shortening hospital stay and so on, thereby providing a new solution for chronic disease management. This article reviews the current application status, mechanism of action and comprehensive benefit of enteral nutrition in the clinical management of chronic diseases. Through systematic review and analysis of existing research findings, the specific application effects and mechanisms of enteral nutrition in chronic disease management are clarified. This review aims to promote the popularization and application of enteral nutrition, in order to effectively improve patients’ treatment outcomes and quality of life, provide scientific evidence for the optimization of clinical management strategies for chronic diseases, and offer theoretical support for the development of enteral nutrition products, and thereby drive the continuous improvement of chronic disease management.

## 1. Introduction

Chronic diseases are characterized by complex etiologies, long latency periods, prolonged disease courses, often resulting in disability or functional impairments, and incomplete curability [1]. With the acceleration of population aging, the advancement of urbanization, and changes in lifestyle, the incidence of chronic diseases is rapidly rising, becoming a significant global public health issue. Chronic diseases are responsible for over 41 million deaths annually, accounting for 74% of total global deaths. Cardiovascular diseases, cancer, chronic respiratory diseases, and diabetes, as the four major types of chronic diseases, respectively cause 17.9 million, 9.3 million, 4.1 million, and 2 million deaths each year, exerting tremendous pressure on the global healthcare system and socio-economy [2,3]. Chronic diseases not only have long treatment cycles and high medical costs, imposing heavy economic burdens on patients’ families and society, but also severely impair patients’ quality of life and limit their ability to participate in society. Therefore, it is particularly important to explore effective chronic disease management strategies to mitigate their negative impact on public health and patients’ quality of life.

Chronic disease patients commonly suffer from issues such as digestive and absorptive disorders, as well as metabolic disturbances. These problems often lead to imbalanced or even deficient nutrient intake, further weakening the patients’ constitution and exacerbating the progression of the diseases. This has become a significant challenge that cannot be ignored in chronic disease management. Clinical studies have shown that enteral nutrition plays a crucial role in improving patients’ nutritional status, promoting recovery, and shortening hospital stays [4,5]. The World Health Organization and other international health agencies consistently emphasize the central role of nutritional intervention in the prevention and management of chronic diseases [6,7,8]. Enteral nutrition, as a form of nutritional support, provides the necessary essential nutrients and other various nutritive substances required for metabolism through the gastrointestinal tract via oral administration or tube feeding [9]. Through scientifically precise nutritional formulations, enteral nutrition aims to meet the specific nutritional needs of chronic disease patients, thereby reducing the risk of malnutrition, enhancing immune function, promoting the recovery of body functions, effectively controlling disease progression, and accelerating the rehabilitation process [10,11]. The application of enteral nutrition provides a new solution for chronic disease management, which is of great significance in delaying disease deterioration, improving patients’ quality of life, and achieving long-term stable control of chronic diseases.

This paper aims to review the application effect and mechanism of enteral nutrition in the clinical management of chronic diseases. By systematically combing and analyzing the existing research results, it elucidates the specific effects and mechanisms of enteral nutrition’s application in managing chronic diseases. It provides scientific support for optimizing chronic disease management strategies and improving patients’ treatment outcomes and quality of life. Furthermore, it offers a theoretical foundation for the research and application of enteral nutrition formulations, facilitates the sustainable advancement of the healthcare industry, and thereby providing valuable insights and references for future research and clinical practice.

## 2. The Application of Enteral Nutrition in the Management of Chronic Diseases

Enteral nutrition is the preferred method for providing nutrition to patients who are unable to meet their daily recommended calorie intake through oral intake, while also helping to maintain the function and integrity of the gastrointestinal system. This method originated in ancient times, with people realizing the feasibility of artificial feeding using simple food formulas, such as eggs, milk, and broth, centuries ago [12]. The modernization of enteral nutrition began in the 19th century, with in-depth research into the macro- and micronutrients in foods, and the establishment of the chemical definition of nutritional formulas in 1949, gradually making enteral nutrition formulas more complex and specialized [13,14]. To date, hundreds of mature and diversified commercial enteral nutrition preparations have emerged on the global market, which not only meet the clinical needs of different patients but also reflect the continuous progress and innovation of enteral nutrition technology [15,16]. In European and American markets, the variety of enteral nutrition preparations is particularly rich. In addition to general-purpose products for ordinary patients, specialized products tailored for patients with specific diseases have also been introduced, such as specific formulations for diabetes, kidney disease, cancer, respiratory diseases, etc. The emergence of these specialized products not only enriches the market supply of enteral nutrition preparations but also provides more precise and effective options for nutritional treatment of patients with different diseases [17,18,19,20,21].

In recent years, an increasing number of clinical studies have demonstrated the significant role of enteral nutrition in the management of chronic diseases [22,23,24,25,26]. Recognizing the importance of enteral nutrition in chronic disease management, international organizations have developed corresponding clinical guidelines or expert consensuses specifically for clinical nutrition management in different diseases and aiming to standardize the clinical application of enteral nutrition. For instance, the European Society for Clinical Nutrition and Metabolism (ESPEN) has published the ESPEN guidelines on nutrition in cancer patients and the ESPEN guideline on Clinical Nutrition in inflammatory bowel disease [27,28]. The American Diabetes Association (ADA) has released Nutrition Therapy for Adults with Diabetes or Prediabetes: A Consensus Report [29]. Additionally, ESPEN and the UK Renal Association have jointly issued the latest guidelines, including the ESPEN practical guideline on clinical nutrition in hospitalized patients with acute or chronic kidney disease as well as Enteral Nutrition in Adults with Chronic Kidney Disease: Things to Consider [30,31]. These authoritative documents all emphasize the importance of enteral nutrition in disease treatment and provide scientific and systematic guidance on nutritional support and primary treatment guidance for healthcare professionals in hospitals and outpatient clinics. Notably, the ESPEN guideline on Clinical Nutrition in inflammatory bowel disease explicitly states that enteral nutrition can be used as primary and supportive nutritional therapy for active inflammatory bowel disease and has been recommended as a first-line treatment for remission in children and adolescents with acute active Crohn’s disease [28]. This fully demonstrates the widespread recognition and application of enteral nutrition in clinical nutrition management. Therefore, as an essential component of chronic disease management, enteral nutrition has broad prospects for development and application and is worth further research and promotion by clinical healthcare professionals.

## 3. The Mechanism of Enteral Nutrition in Clinical Nutritional Management for Chronic Diseases

### 3.1. Cancer

Enteral nutrition plays an indispensable role in the clinical nutritional support of cancer patients. Cancer patients often suffer from problems. such as decreased appetite, impaired digestion and absorption function, metabolic abnormalities, and exacerbated nutritional depletion, as a result of treatments like surgical procedures, chemotherapy, and radiotherapy [32]. Enteral nutrition provides comprehensive and balanced nutritional support, particularly rich in key nutrients, such as high-quality proteins, essential fatty acids, vitamins, and minerals, which helps maintain or improve the nutritional status of patients, enhancing the body’s tolerance and response capability to treatment [33]. Clinical studies have shown that the implementation of enteral nutrition support during perioperative period or chemoradiotherapy can significantly elevate the nutritional levels of cancer patients, strengthen immune function, and reduce inflammatory responses (Table 1). Taking colon cancer patients undergoing chemotherapy as an example, compared with parenteral nutrition, the levels of serum hemoglobin, albumin, prealbumin, and immunoglobulin A, G, and M were significantly increased in patients treated with enteral nutrition, while the levels of inflammatory factors, such as interleukin-1, interleukin-8, and tumor necrosis factor-α, were decreased, thus improving the quality of life and correspondingly decreasing the incidence of adverse reactions [34]. Further study has also reached a similar conclusion in postoperative patients with liver cancer, revealing that enteral nutrition can reduce the levels of inflammatory cytokines interleukin-1, interleukin-6, and cancer necrosis factor-α, while increasing the expression of immune factors CD4^+^ and CD8^+^, thus contributing to the recovery of patients [35]. For lung cancer patients undergoing chemotherapy, enteral nutrition support can also significantly improve nutritional status and lung function, enhancing the quality of life of patients [36]. Additionally, the combined application of enteral nutrition and microbial preparations has demonstrated promising clinical outcomes. In the intestinal preparation of elderly patients with colorectal cancer, the use of enteral nutrition combined with microbial preparations not only alleviated systemic inflammatory responses and improved nutritional status, but also reduced the risk of postoperative complications, promoting rapid recovery of patients [37]. It is worth noting that a study on nutrition management of patients with esophageal cancer after discharge indicates that continuous oral enteral nutrition preparations can significantly improve patients’ subjective global evaluation, hemoglobin, serum albumin and a number of immune indicators compared with conventional diet, emphasizing the importance of home enteral nutrition in improving patients’ immune function [38]. Apart from improvements in biochemical indicators, enteral nutrition has also been found to improve skeletal muscle mass and increase body mass index (BMI) [38,39,40]. One of the studies on esophageal cancer found that enteral nutrition support inhibited skeletal muscle mass loss in patients with esophageal cancer compared to parenteral nutrition support [39]. In summary, early and regular enteral nutrition support has significant clinical significance in improving the nutritional status of cancer patients, enhancing immune function, reducing the incidence of adverse reactions and improving quality of life, and is worthy of widespread promotion in clinical practice.

### 3.2. Kidney Disease

Enteral nutrition plays a crucial role in clinical nutritional management for patients with kidney disease. Its mechanism of action mainly lies in providing precise nutritional support according to the specific nutritional needs of patients with kidney disease, including strict control of the intake of key nutrients, such as protein, phosphorus, potassium, and sodium, thereby reducing the burden on the kidneys, regulating electrolyte balance, and maintaining the stability of calcium and phosphorus metabolism [66,67]. Protein-energy wasting (PEW), characterized by low levels of serum albumin or prealbumin, muscle loss, and weight reduction, is a common and highly predictable indicator of poor prognosis in patients with chronic kidney disease [68]. Enteral nutrition, as an effective intervention strategy, has been widely proven to alleviate or even reverse the state of PEW, thus exerting a positive impact on the patients’ nutritional status and disease prognosis [69,70,71]. For instance, oral nutritional supplements specifically designed for kidney diseases can significantly increase serum albumin levels in malnourished patients with chronic kidney disease, reduce the dosage of erythropoietin required, and optimize anthropometric parameters [44]. Moreover, enteral nutrition support has shown significant benefits in the short-term treatment of patients with acutely impaired kidney function. Through enteral nutrition support, the key nutritional indicators, such as serum albumin, prealbumin, transferrin, and lymphocyte count, have shown significant improvements [72]. For children with chronic kidney disease, malnutrition and growth retardation are the most common complications and important indicators of poor prognosis, and their growth is crucial for long-term prognosis, including final adult height and cognitive function. The application of enteral nutrition has brought significant growth and development benefits, especially in improving the height and weight of pre-adolescent children [73]. A study has shown that children with chronic kidney disease and hyperkalemia experienced improved growth rates after receiving adult renal formulas, which were well-tolerated and effectively reduced potassium exposure [74]. In summary, enteral nutrition, by precisely meeting the nutritional needs of patients with kidney diseases and effectively regulating the intake of key nutrients, has significantly improved patients’ nutritional status, effectively delayed the progression of the disease, and improved patients’ quality of life.

### 3.3. Diabetes

Hyperglycemia is a major challenge for patients with diabetes, particularly in the context of enteral feeding, where newly diagnosed hyperglycemia is even considered an independent prognostic factor for death in such patients [75,76]. Hyperglycemia not only affects the quality of life of diabetic patients, but also may have profound impacts on wound healing, length of hospital stay, and the risk of complications in hospitalized patients [77,78]. Enteral nutrition plays a crucial role in the management of diabetic patients. It provides patients with the necessary macronutrients and micronutrients, including partial or total energy, proteins, vitamins, and minerals, thereby reducing the risk of malnutrition. Specifically, specialized enteral nutrition formulas for diabetic patients contain specific ingredients, such as low glycemic index (low-GI) carbohydrates (e.g., fructose) and abundant monounsaturated fatty acids, aimed at better controlling postprandial glucose levels [47,79,80,81]. Scientific and reasonable selection of enteral nutrition formulas is essential for maintaining stable blood glucose levels, promoting body recovery, and reducing the risk of complications in diabetic patients during enteral feeding. A study evaluating the application effects of a specific high-protein, high-calorie enteral nutrition formula in diabetic patients found that it significantly reduced the incidence of malnutrition (from 78.6% to 29.9%), accompanied by significant improvements in nutritional indicators, such as weight, BMI, albumin, prealbumin, and transferrin, as well as a significant decrease in CRP levels and the CRP/albumin ratio [48]. Furthermore, this formula also significantly lowered blood glucose and glycated hemoglobin (HbA1c) levels, with good gastrointestinal tolerance, a low incidence of moderate-to-severe symptoms, and stability during follow-up. Compared with standard enteral nutrition formulas, diabetes-specific formulas demonstrated significant advantages in lowering blood glucose levels, reducing insulin requirements, and decreasing the risk of acquired infections [49]. Another study similarly validated the effectiveness of diabetes-specific enteral nutrition formulas in improving glycemic control in patients with type 2 diabetes, showing that high-protein, low-carbohydrate enteral nutrition formulas can significantly improve glycemic control in these patients [50]. A clinical nutrition study on radical gastrectomy for gastric cancer patients with diabetes found that enteral nutrition not only provided sufficient nutritional support but also significantly stabilized blood glucose levels, reduced the incidence of postoperative complications, and effectively shortened hospital stay [51]. This finding provides strong evidence for the clinical application of enteral nutrition in diabetic patients with surgical diseases and is worth widely promoting and applying in clinical practice. Overall, enteral nutrition, by providing specific nutritional support, can effectively control blood glucose levels in diabetic patients, improve their nutritional status, reduce the risk of complications, and facilitate physical recovery. Compared with standard formulas, diabetes-specific formulas offer significant advantages in lowering blood glucose, insulin requirements, and infection risks.

### 3.4. Inflammatory Bowel Disease

Inflammatory Bowel Disease (IBD), which includes Crohn’s Disease (CD) and Ulcerative Colitis (UC), is a complex chronic intestinal inflammatory disorder that is becoming a global concern. IBD patients are at risk of malnutrition due to reduced food intake, malabsorption, increased gastrointestinal losses, increased energy demands due to hyper-catabolism, and drug–nutrient interactions [82]. Clinical studies have shown that malnutrition not only affects patient prognosis, but also increases the incidence of complications and mortality, and severely diminishes the quality of life [28]. The effectiveness of enteral nutrition in the treatment of inflammatory bowel disease has been proven. In the treatment of IBD, enteral nutrition has emerged as an effective method for inducing remission, particularly in the management of Crohn’s Disease, and its effectiveness is second only to that of corticosteroids [83,84]. Studies have demonstrated that enteral nutrition support not only improves nutritional indicators, such as serum albumin, prealbumin, and hemoglobin levels, but also significantly reduces markers of inflammation, including *C*-reactive protein and fecal calprotectin levels [52,53,54,55,56]. More importantly, enteral nutrition also significantly decreases CDAI, promotes weight gain, and improves intestinal mucosal healing [52,55,56]. Notably, enteral nutrition also induces positive changes in the gut microbiota, manifested by a significant increase in microbial α-diversity, with beneficial bacteria, such as Firmicutes, Ruminococcus, Lachnospiraceae, and Faecalibacterium prausnitzii, significantly increasing, while harmful bacteria, such as Escherichia/Shigella, Dialister invisus, Haemophilus, and Streptococcus, significantly decrease [53,54,57,60]. These beneficial bacteria play a crucial role in inhibiting the expression of inflammatory cytokines, regulating the body’s immune response, maintaining intestinal barrier function, and promoting mucosal repair [85,86]. For example, Faecalibacterium prausnitzii, which can produce short-chain fatty acids, plays an important role in regulating cytokine release, maintaining intestinal mucosal barrier function, modulating intestinal immune responses, and providing energy for intestinal epithelial cells [87,88,89]. Therefore, as a means of clinical nutrition treatment, the core mechanism of enteral nutrition is to optimize the nutritional status of IBD patients, reduce the inflammatory response of the body, enhance the immunity of the body, accelerate the repair process of damaged intestinal tissues, improve intestinal dysbiosis, and thereby effectively alleviate intestinal inflammation.

### 3.5. Chronic Respiratory Disease

Chronic respiratory diseases, particularly chronic obstructive pulmonary disease (COPD), are a serious global problem. COPD is the third leading cause of death worldwide and has become one of the major challenges in public health [90]. Patients with COPD often experience increased energy consumption, decreased appetite, and insufficient nutrition intake due to long-term airway obstruction and dyspnea, which can lead to malnutrition and subsequently weakened immune function [91]. Weight loss caused by malnutrition is a risk factor for poor prognosis in patients with advanced COPD [21,92,93]. Clinical studies have demonstrated that enteral nutrition support exhibits significant therapeutic effects in patients with COPD. Enteral nutrition not only markedly improves the nutritional and inflammatory indicators in COPD patients requiring mechanical ventilation, but also enhances the diaphragmatic function, thereby improving clinical outcomes and patient prognosis [61,62]. Furthermore, in response to the common decline in muscle function and lung function in COPD patients, enteral nutrition support has also shown a positive effect, significantly increasing the grip strength and forced expiratory volume in one second (FEV1), thereby improving the muscle and lung function of patients [63]. Similarly, another study suggests that early standardized enteral nutrition support can prevent acute muscle loss in COPD patients [64]. In addition, as an adjunct to drug therapy, enteral nutrition can significantly enhance the therapeutic effect of drugs. Compared with drug therapy alone, enteral nutrition support combined with drug therapy not only greatly improves the nutritional status and inflammation level of patients, but also significantly improves the immune function of patients and significantly enhances their lung function [60]. In addition, clinical studies have also demonstrated that, compared with conventional nutritional support, high-fat, low-carbohydrate enteral nutrition therapy can more adequately meet the body’s oxygen consumption needs, effectively decrease CO_2_ production, and significantly enhance lung function. This indicates that COPD-special enteral nutrition support has a superior effect in clinical practice [94]. In summary, enteral nutrition comprehensively improves the clinical outcomes and prognosis of COPD patients through multiple mechanisms, including optimizing nutritional status, alleviating inflammation, enhancing diaphragm and muscle strength, improving pulmonary function, and assisting in pharmacotherapy, thereby significantly enhancing the quality of life of patients and providing strong support for the treatment of COPD.

## 4. Comprehensive Benefit Analysis of Enteral Nutrition in the Management of Chronic Diseases

**Figure 1:**
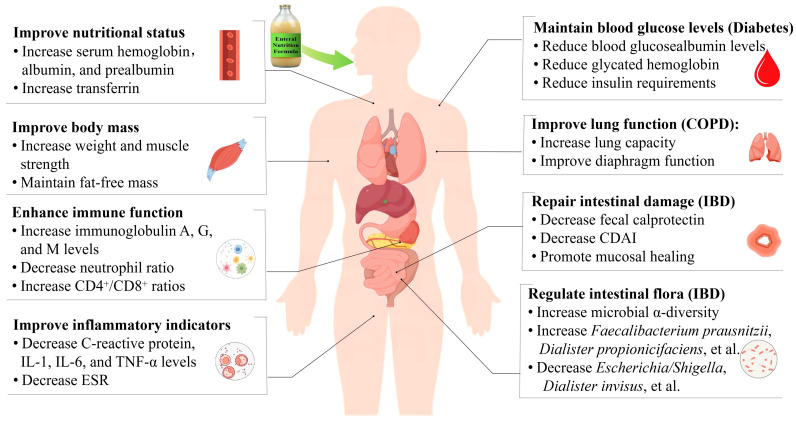
The action of mechanisms of enteral nutrition in chronic disease management (by Figdraw, https://www.figdraw.com/static/index.html#/, 1 December 2024). Abbreviations used: CDAI, Crohn’s disease activity index; COPD, chronic obstructive pulmonary disease; ESR, erythrocyte sedimentation rate; IBD, inflammatory bowel disease; IL-1, interleukin 1; IL-6, interleukin 6; TNF-α, tumor necrosis factor-α.

Enteral nutrition has shown significant comprehensive benefits in the clinical management of chronic diseases. It not only excels in controlling disease and reducing complications, but also greatly improves patients’ quality of life and effectively reduces medical costs. In terms of clinical outcomes, as illustrated in Table 1 and Figure 1, clinical trials and observational studies have consistently shown that enteral nutrition support positively impacts symptom improvement, disease control, and complication prevention in patients with chronic diseases. Particularly, enteral nutrition preparations designed for specific diseases, such as diabetes, respiratory diseases, kidney diseases, and other chronic diseases, exhibit exceptional clinical efficacy. These specific formulations effectively alleviate and improve disease symptoms by precisely delivering the nutrients required by patients to meet their specific nutritional and metabolic needs.

**Figure 2:**
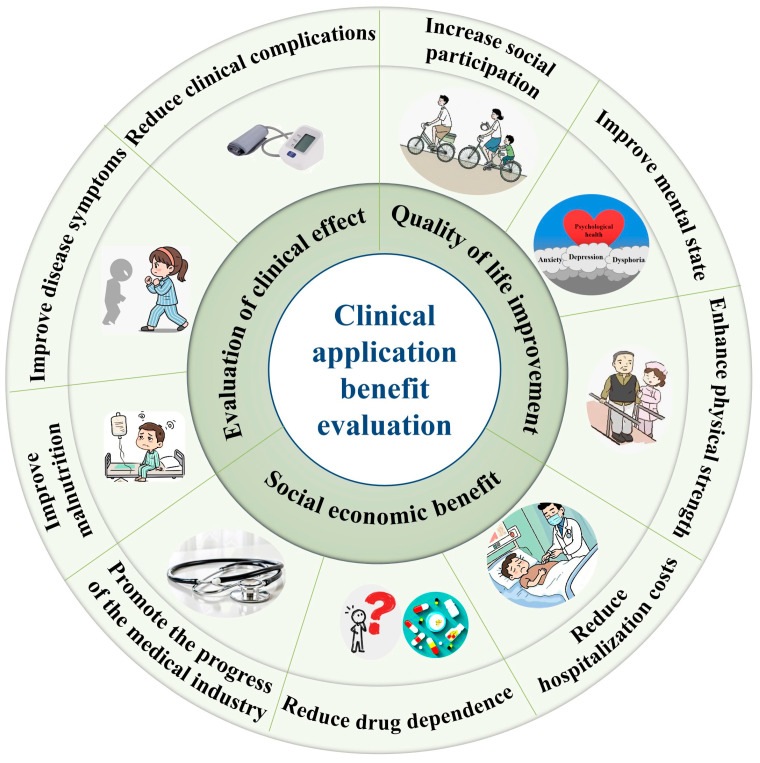
Comprehensive benefits of enteral nutrition in the management of chronic diseases.

In terms of improving the quality of life, enteral nutrition helps patients strengthen their physical strength and improve their ability to perform daily activities by providing comprehensive nutritional support (Table 1, Figure 2). This not only assists patients in better coping with the challenges of disease, but also elevates their quality of life. Furthermore, enteral nutrition can also effectively reduce the psychological burden on patients [95,96,97,98]. Many chronic disease patients experience anxiety, depression, and other psychological issues during long-term treatment. Enteral nutrition, as an effective adjuvant therapy, can alleviate the symptoms of patients, boost their treatment confidence, and thereby improve their psychological state [99]. Additionally, enteral nutrition can further improve the social function of patients by improving their nutritional status, so that patients can better integrate into society, participate in social interaction, and further enhance their quality of life [100,101].

From the perspective of economic benefits, the application of enteral nutrition in the clinical management of chronic diseases also offers notable advantages. On the one hand, enteral nutrition can reduce complications and mortality rates, thereby shortening hospital stays and reducing hospitalization costs [102,103,104,105]. Consequently, this brings substantial economic relief to patients’ families and the healthcare system. Taking the application of diabetes-specific enteral formulations in intensive care unit (ICU) patients with type 2 diabetes as an example, the study showed that, compared with patients who did not receive the formulations, patients who received the formulations had significantly lower mortality rates, a 9.3% reduction in insulin requirements, and a 27.2% reduction in medical expenditures, and significant savings were also observed in ICU costs, examination costs, and nutritional support expenses [103]. On the other hand, the long-term application of enteral nutrition support can also reduce patients’ dependence on high-priced drugs and treatments. Through comprehensive and balanced nutritional support, patients’ nutritional status and immunity are doubly enhanced, thus reducing the reliance on drugs and treatments, and further saving medical expenses. For instance, enteral nutrition as induction therapy is second only to or even superior to glucocorticoids in effectiveness in Crohn’s disease, and its application significantly reduces the dependence of patients on drugs [84,106]. Furthermore, the promotion and application of enteral nutrition will also drive innovation and development in the field of chronic disease management. With continuous progress in technology and the upgrading of products, more chronic disease patients will benefit, thereby promoting the vigorous development of the medical and health industry.

## 5. Challenges and Strategies for Enteral Nutrition in Chronic Disease Management

In the comprehensive system of chronic disease management, enteral nutrition, as an important nutritional support strategy, faces multifaceted challenges. Poor patient adherence is a core issue that directly impacts the effectiveness of clinical interventions [107,108]. Although enteral nutrition can provide precise nutritional support and effectively meet the special needs of patients with chronic diseases, many patients have limited understanding of it, viewing it merely as a supplementary means without fully recognizing its importance as a complement to drug therapy or even as an independent treatment under certain conditions. Additionally, concerns about taste and ease of use further reduce patient adherence [109,110]. In response to this challenge, we can adopt a series of effective strategies to improve patient compliance. One key approach is to strengthen patient education and training by organizing professional lectures and distributing science popularization materials, and other means, in order to enhance patients’ scientific understanding of enteral nutrition. Additionally, establishing patient communication platforms to encourage patients to share their experiences and outcomes regarding enteral nutrition can enhance their confidence and trust through real-life cases. Furthermore, developing enteral nutrition products with good taste and tolerance is also an important way to improve adherence.

In clinical management practice, enteral nutrition complications are also an important problem that cannot be ignored. Especially when patients receive home enteral nutrition support, it is often difficult to obtain timely and professional medical assistance [111,112,113]. The occurrence of enteral nutrition complications is also one of the important factors leading to poor compliance. These complications mainly include tube-related complications (such as tube clogging or obstruction, stomal leakage, cutaneous fistulas, etc.), infection risks, metabolic disorders, liver dysfunction, aspiration pneumonia and digestive intolerances (nausea, vomiting, abdominal pain, diarrhea, etc.), with digestive intolerances being the most common issues [18,114,115,116]. Once patients experience related complications, this may cause serious consequences [117,118]. Therefore, it is necessary to pay close attention to the situation of patients and take effective intervention measures quickly to ensure safety and health. In dealing with enteral nutrition complications, multiple factors, such as formula selection, infusion route, feeding mode, dosing frequency, dosing time, and dosing amount, should be comprehensively considered [116]. Medical staff should strictly implement aseptic procedures, closely monitor electrolyte level and liver function indicators, and ensure the safety and effectiveness of enteral nutrition support. At the same time, the professional knowledge training of patients and medical staff should be strengthened to improve the ability to respond to emergencies [119,120]. In addition, recent studies have shown that new tube feeding protocols (such as nasal bridles) can be used to address tube-related complications, and peptide-based enteral nutrition formulations can be used to reduce digestive intolerance, which provides valuable exploration and practice paths for the continuous optimization of enteral nutrition support [121,122,123,124].

Moreover, precise matching of the diversity of enteral nutrition products to patients’ individualized needs is also a significant challenge [125,126]. There are many types of chronic diseases and different stages of disease development, a situation which requires that enteral nutrition preparations must be able to accurately meet the specific nutritional needs of different patients. However, the current market offers a relatively limited variety of enteral nutrition products, making it difficult to meet the individualized needs of most patients. This mismatch between supply and demand not only increases the difficulty for patients in selecting suitable products but may also result in some patients being unable to obtain nutrition support that is fully compatible with their condition, thereby limiting the widespread application and full efficacy of enteral nutrition in chronic disease management. In view of the diversity and individual needs of enteral nutrition products, we should vigorously promote product research and innovation. Enterprises should increase investment in scientific research and commit to developing a wider variety of higher-quality enteral nutrition products to meet the special nutritional needs of different chronic disease patients. At the same time, enterprises should actively explore personalized customization services, providing precise customization based on patients’ specific conditions and nutritional needs to achieve true personalized nutritional support. Moreover, governments and relevant institutions should also introduce relevant policies to encourage enterprises to enhance research and development as well as production of enteral nutrition products, promote rapid industrial development, and provide more diversified and more precise nutritional solutions for chronic disease management, thereby helping the rehabilitation and health management of patients with chronic diseases.

## 6. Conclusions

Enteral nutrition, as a scientific and precise nutritional intervention method, plays an irreplaceable role in the management of chronic diseases. By providing comprehensive and balanced nutritional support, enteral nutrition can meet the special nutritional needs of chronic disease patients, improve their nutritional status, enhance immune function, promote the recovery of physical functions, and then effectively control the progression of the disease and accelerate the rehabilitation process. Furthermore, the application of enteral nutrition also brings significant economic benefits. By reducing complications and mortality, shortening hospital stays, and decreasing dependence on expensive drugs and treatments, enteral nutrition brings substantial economic relief for patients and their families, while also reducing the burden on the healthcare system.

Clinical practice has demonstrated that enteral nutrition not only shows significant clinical effects and improvements in quality of life in cancer, kidney disease, diabetes, inflammatory bowel disease, and chronic respiratory diseases, but also exhibits numerous benefits in other chronic diseases, such as neurodegenerative diseases and autoimmune diseases [127,128]. Although sufficient research data on the specific effects and application strategies of enteral nutrition are lacking for some chronic diseases, this is not enough to obscure its enormous potential and value in the management of chronic diseases.

In the future, we should strengthen in-depth research and extensive application of enteral nutrition in various chronic diseases, continuously expand its application fields, and fully tap its potential value. At the same time, we also look forward to more research focusing on the long-term effects and impacts of enteral nutrition in the management of chronic diseases, providing more comprehensive insights and references for clinical practice, thereby continuously optimizing chronic disease management strategies and promoting the sustained development of the healthcare industry. With the increasing awareness of patient nutrition and the continuous progress of enteral nutrition technology and product iteration, we believe that more and more patients with chronic diseases will benefit from enteral nutrition.

## Figures and Tables

**Table 1:** The application of enteral nutrition in the clinical management of patients with chronic diseases.

Countries/Regions	Disease Type	Type of Clinical Study Design	Intervention Time	Intervention Method	Conclusion	Reference
Poland	Oropharyngeal cancer and digestive system cancer	Prospective, observational study	3 months	Enteral nutrition	Enteral nutrition can effectively maintain body mass (weight, triceps skinfold thickness), composition (fat-free mass, body cell mass), and nutritional status (albumin levels).	[40]
Turkey	Abdominal cancer	Randomized, controlled trial	37 days	Immunomodulatory enteral nutrition	Immunomodulatory enteral nutrition can reduce the incidence of surgical site infections, pneumonia, urinary tract infections, and shorten the length of hospital stay in patients undergoing abdominal tumor surgery.	[41]
Japan	Esophageal cancer	Randomized, open-label clinical trial.	15 days	Enteral nutrition rich in ω3 fatty acids	The administration of enteral nutrition rich in ω3 fatty acids has been shown to reduce the frequency of chemotherapy-induced mucosal toxicity, such as stomatitis and diarrhea, and exhibits a hepatoprotective effect during chemotherapy.	[42]
China	Colon Cancer	Double-blind random sampling approach.	6 months	Enteral nutrition	The levels of serum hemoglobin, albumin, prealbumin, immunoglobulin A, G, and M in the enteral nutrition group were significantly increased. Conversely, the levels of serum interleukin-1, interleukin-8, and tumor necrosis factor-α were decreased.	[34]
China	Liver cancer	Retrospective study	3 days	Enteral nutrition	The differences in CD4^+^, CD8^+^, interleukin-1, interleukin-6, tumor necrosis factor-α, the time to first flatulence, and the time to first defecation were all statistically significant.	[35]
China	Lung cancer	Randomized, controlled trial	2 weeks	Enteral nutrition	Increases in body weight, triceps skin fold thickness (TSP), mid-arm muscle circumference (MAMC), total protein, albumin, and hemoglobin were observed, along with decreases in white blood cell count and the incidence of adverse reactions.	[36]
China	Colorectal Cancer	Randomized, controlled trial	3 days	Enteral nutrition	The total white blood cell count, neutrophil ratio, *C*-reactive protein levels, IL-6 levels, and postoperative complication rates were significantly lower than those in the control group, while the serum albumin, prealbumin, and transferrin levels were superior to those in the control group.	[37]
China	Esophageal cancer	Randomized, controlled trial	8 weeks	Enteral nutrition	The Enteral nutrition group had significantly higher Body Mass Index (BMI), Scored Patient-Generated Subjective Global Assessment (PG-SGA) scores, serum albumin levels, serum prealbumin levels, CD4 and CD8 T-cell counts, CD4/CD8 ratios, immunoglobulin A, G, and M levels compared to the control group.	[38]
Japan	Esophageal cancer	Randomized, controlled trial	17 days	Enteral nutrition	Enteral nutrition supports the inhibition of skeletal muscle mass loss in esophageal cancer patients during neoadjuvant chemotherapy.	[39]
Italy	Upper gastrointestinal cancer	Multicentre randomised clinical trial	6 months	Enteral nutrition	Helps maintain weight without any safety issues or negative impacts on quality of life.	[43]
Turkey	Chronic Kidney Disease	Randomized, controlled trial	6 months	Renal-specific oral nutritional supplement	Renal-specific oral nutritional supplement improved patients’ serum albumin levels and anthropometric indicators, and reduced the dose of erythropoietin.	[44]
China	Chronic Kidney Disease	Prospective, multicenter, single-arm, and open-label study	6 months	Renal-specific oral nutritional supplement	Renal-specific oral nutritional supplement increased patients’ energy intake and maintained their serum albumin levels, nutritional status, and quality of life. Their body weight and grip strength significantly increased, while the glomerular filtration rate slightly decreased.	[45]
USA	Kidney Disease	Randomized crossover design trial	12 months	Enteral nutrition	Improved serum nutrition indicators, resulting in reduced hospital admission frequency and length of stay.	[46]
The Netherlands	Type 2 diabetes mellitus	Randomized, controlled, double-blind, cross-over study	1 day	Specific high-protein, high-calorie enteral nutrition formula	The use of diabetes-specific formulas can significantly improve the 24-h and postprandial blood glucose levels in diabetic patients.	[47]
Spain	Diabetes	Multicenter, prospective, observational, real-life study	24 weeks	Specific high-protein, high-calorie enteral nutrition formula	The use of a specific high-protein, high-calorie enteral nutrition formula resulted in a decrease in the proportion of malnourished patients from 78.6% to 29.9%. Blood glucose and glycated hemoglobin levels were significantly reduced, while weight, BMI, albumin, prealbumin, and transferrin levels were significantly increased. *C*-reactive protein levels were significantly decreased, and the *C*-reactive protein/albumin ratio was reduced. Gastrointestinal tolerance was good, with only a few patients experiencing moderate to severe symptoms.	[48]
Spain	Diabetes	Prospective, open-label, blind-randomized, multicenter study	4 weeks	Diabetes-specific formula	Compared with the standard control group, the diabetes-specific formula significantly reduced insulin requirements, blood glucose levels, capillary blood glucose levels, and the incidence of ventilator-associated tracheobronchitis or pneumonia.	[49]
USA	Type 2 diabetes mellitus	Randomized crossover trial	4 h	High-protein and low-carbohydrate enteral nutrition formula	A high-protein and low-carbohydrate enteral nutrition formula can significantly improve glycemic control in patients with type 2 diabetes, without significant effects on insulin response.	[50]
China	Gastric cancer complicated with diabetes mellitus	Randomized, controlled trial	8 days	Enteral nutrition	Early enteral nutrition support helps patients maintain good nutritional status, reduces postoperative complications, stabilizes blood glucose levels, facilitates earlier postoperative mobilization, shortens hospital stays, and lowers costs.	[51]
Sweden	Crohn’s Disease	Prospective cohort study	6 weeks	Enteral nutrition	The erythrocyte sedimentation rate (ESR), *C*-reactive protein, and fecal calprotectin are significantly decreased, while hemoglobin, albumin levels, and body weight are significantly increased. Colonoscopy shows promotion of mucosal healing.	[52]
China	Crohn’s Disease	Retrospective cohort study	21 weeks	Enteral nutrition	Preoperative serum levels of albumin, prealbumin, and hemoglobin are elevated, and the incidence of postoperative complications is reduced.	[53]
New Zealand	Crohn’s Disease	Prospective non-randomized pilot study	8 weeks	Enteral nutrition	The levels of inflammatory markers *C*-reactive protein and fecal calprotectin decrease, while the levels of nutritional markers serum insulin-like growth factor 1 (IGF-1) and albumin increase.	[54]
Australian	Crohn’s Disease	Retrospective analysis	6 weeks	Enteral nutrition	C-reactive protein levels decrease, serum albumin levels increase, body weight increases, and the need for surgical intervention as well as postoperative complications are reduced.	[55]
China	Crohn’s Disease	Prospective cohort study	12 weeks	Enteral nutrition	CDAI, *C*-reactive protein, ESR, and platelet counts are significantly reduced, while albumin and hemoglobin levels are increased. Colonoscopy shows promotion of mucosal healing.	[56]
Canada	Inflammatory Bowel Disease	-	10 weeks	Enteral nutrition	*Faecalibacterium prausnitzii*, *Dialister propionicifaciens*, and *Parabacteroides merdae* are significantly increased, while *Escherichia/Shigella*, *Dialister invisus*, and *Negativibacillus* are significantly decreased. Fecal microbial α-diversity is also significantly increased.	[57]
Canada	Inflammatory Bowel Disease	Prospective cohort study	8 weeks	Enteral nutrition	The abundance of *Blautia*, *Sellimonas*, and uncharacterized bacteria from the family *Ruminococcaceae* increases, while the abundance of *Granulicatella*, *Haemophilus*, and *Streptococcus* decreases.	[58]
China	Crohn’s Disease	Prospective single-center cohort study	8 weeks	Enteral nutrition	The Pediatric Crohn’s Disease Activity Index (PCDAI) score and calprotectin levels decrease, while the microbiome and bile acid metabolism return to normal levels. The relative expression of *Firmicutes phylum*, *Flavonifractor*, and *Clostridium V* increases.	[59]
China	Crohn’s Disease	Cohort Study	8 weeks	Enteral nutrition	ESR, *C*-reactive protein, and CDAI significantly decrease, while serum albumin levels increase. The abundance of *Firmicutes*, *Ruminococcus*, *Lachnospiraceae*, *Anaerotruncus*, *Flavonifractor*, and *Novosphingobium* significantly increases, while the abundance of *Proteobacteria* decreases.	[60]
China	Chronic Obstructive Pulmonary Disease	Randomized, controlled trial	14 days	Enteral nutrition	Significantly improves the nutritional status and diaphragmatic function of patients, inhibits inflammatory responses, shortens the duration of mechanical ventilation, and enhance clinical treatment efficacy and prognosis.	[61]
China	Chronic Obstructive Pulmonary Disease	Randomized, controlled trial	-	Enteral nutrition	Improves patients’ energy metabolism and alleviates respiratory muscle fatigue during and after weaning from mechanical ventilation, without increasing the incidence of related complications.	[62]
Turkey	Chronic Obstructive Pulmonary Disease	Rospective, controlled, randomized trial	8 days	Enteral nutrition	Increased patients’ grip strength and forced expiratory volume in second (FEV1).	[63]
China	Chronic Obstructive Pulmonary Disease	Observational study	28 days	Enteral nutrition	Early standardized enteral nutrition can prevent acute muscle loss and intensive care unit-acquired weakness (ICU-AW) in patients with acute exacerbations of chronic obstructive pulmonary disease (AECOPD).	[64]
China	Chronic Obstructive Pulmonary Disease	Randomized, controlled trial	4 weeks	Enteral nutrition	Compared with the control group, patients in the enteral nutrition group showed significant improvements in partial pressure of carbon dioxide, forced expiratory volume in one second/forced vital capacity, and partial pressure of oxygen. The levels of immunoglobulin A, G, and M, as well as the number of CD4^+^/CD8^+^ and CD4^+^/CD3^+^ T cells were higher in the EN group than in the control group. Additionally, compared with the control group, the enteral nutrition group had increased levels of inflammatory factors, such as tumor necrosis factor-α and interleukin-1 β, while the level of IL-6 was decreased. The serum total protein, albumin, and transferrin levels were significantly higher in the enteral nutrition group than in the control group.	[65]
